# Changes in Renal Function and Oxidative Status Associated with the Hypotensive Effects of Oleanolic Acid and Related Synthetic Derivatives in Experimental Animals

**DOI:** 10.1371/journal.pone.0128192

**Published:** 2015-06-05

**Authors:** Hlengiwe Pretty Madlala, Fanie Retief Van Heerden, Kanigula Mubagwa, Cephas Tagumirwa Musabayane

**Affiliations:** 1 School of Laboratory Medicine and Medical Sciences, College of Health Sciences, University of KwaZulu-Natal, Durban, South Africa; 2 School of Chemistry and Physics, College of Science, Engineering and Agriculture, University of KwaZulu-Natal, Pietermaritzburg, South Africa; 3 Department of Cardiovascular Sciences, Faculty of Medicine, Katholieke Universiteit Leuven, Leuven, Belgium; Max-Delbrück Center for Molecular Medicine (MDC), GERMANY

## Abstract

**Purpose:**

The triterpene oleanolic acid (OA) is known to possess antihypertensive actions. In the present study we to compared the effects of the triterpene on mean arterial blood pressure (MAP) and kidney function following acute administration in normotensive animals with those of its related oleanane synthetic derivatives (brominated oleanolic acid, Br-OA and oleanolic acid methyl ester, Me-OA). We also used experimental models of hypertension to further explore the effects of sub-chronic oral OA treatment and evaluated influences on oxidative status.

**Methods:**

OA was extracted from dried flower buds of *Syzygium aromaticum* using a previously validated protocol in our laboratory. Me-OA and Br-OA were synthesized according to a method described. Rats were supplemented with lithium chloride (12 mmol L^-1^) prior to experimentation in order to raise plasma lithium to allow measurements of lithium clearance and fractional excretion (FE_Li_) as indices of proximal tubular Na^+^ handling. Anaesthetized animals were continuously infused via the right jugular with 0.077M NaCl. MAP was measured via a cannula inserted in the carotid artery, and urine was collected through a cannula inserted in the bladder. After a 3.5 h equilibration, MAP, urine flow, electrolyte excretion rates were determined for 4 h of 1 h control, 1.5 h treatment and 1.5 h recovery periods. OA, Me-OA and Br-OA were added to the infusate during the treatment period. We evaluated sub-chronic effects on MAP and kidney function in normotensive Wistar rats and in two animal models of hypertension, spontaneously hypertensive rats (SHR) and Dahl salt-sensitive (DSS) rats, during 9-week administration of OA (p.o.). Tissue oxidative status was examined in these animals at the end of the study. Increasing evidence suggests that and renal function disturbances and oxidative stress play major roles in the pathogenesis of hypertension.

**Results:**

Acute infusion OA and oleanane derivatives displayed qualitatively similar effects in decreasing MAP and increasing urinary Na^+^ outputs. The drugs increased the FE_Na_ and FE_Li_ without influencing GFR indicating that at least part of the overall natriuretic effect involved proximal tubular Na^+^ reabsorption. Sub-chronic OA administration (p.o.) also elicited hypotensive responses in Wistar, DSS and SHR rats. The MAP lowering effect was more marked in hypertensive animals and were positively correlated with increased urinary Na^+^ excretion. Compared with respective control rats, OA treatment reduced malondialdehyde (MDA, a marker of lipid peroxidation) and increased activities of antioxidant enzymes; superoxide dismutase and glutathione peroxidase in hepatic, cardiac and renal tissues.

**Conclusions:**

OA and oleanane derivatives have similar effects on MAP, kidney function and oxidative stress. The amelioration of oxidative stress and blood pressure lowering effects by OA are more marked in hypertensive animals and correlated with an increased urinary Na^+^ output.

**Novelty of the Work:**

The results of this study are novel in that they show 1) a correlation between blood pressure reduction and increased urinary Na^+^ excretion by OA, 2) a more marked MAP reduction in hypertensive animals and 3) a drug-induced decrease in proximal tubule Na^+^ reabsorption. The results may also be clinically relevant because OA is effective via oral administration.

## Introduction

Hypertension continues to be globally responsible for approximately 9.4 million deaths each year [1] despite the various conventional drugs that are available to treat the disease. The high mortality can partly be attributed to side effects of available drugs or inaccessibility to conventional drug treatments to communities from poor socioeconomic background because of their relative high cost [2]. This problem has resulted in growing interest in the use of medicinal plant products because they are considered to be cheap and are easily accessible to the general population in developing countries [3]. Medicinal plant remedies and their derivatives should ideally demonstrate their efficacy and tolerability following the same rules used for synthetic drugs. Scientific research has only recently begun to provide evidence for the mechanisms underlying their therapeutic and pharmacological effects, although ethnomedical use of medicinal plant extracts by different cultures around the world dates back to many centuries. In search for plants with therapeutic properties for the treatment of hypertension and complications, our laboratory has scientifically evaluated several plant species [4,5]. In particular, we have isolated triterpenes oleanolic acid (OA) [6–9] and maslinic acid (MA) [10] from *Syzygium* spp and focused on the therapeutic effects of OA.

Literature has reported diverse pharmacological properties of OA and this therapeutic importance has led to the use of this triterpene as a starting material for the synthesis of new derivatives [11]. In comparison to OA, the new synthetic compounds are reported to have improved biological activities [12–14]. Therefore, we synthesized two oleanane derivatives, a methyl ester (Me-OA) and a brominated derivative of OA (Br-OA), and investigated their hypotensive effects in experimental animals. We have previously reported that OA exhibits blood pressure lowering effects in normotensive animals, but the underlying mechanisms were not determined [7]. In the present study we used Dahl-salt sensitive (DSS) and spontaneously hypertensive rats (SHR) to test their responsiveness to OA in comparison with non-hypertensive controls. DSS and SHR rats are accepted experimental models of hypertension emphasizing the roles of diet and genetic factors, respectively [15]. The mechanisms for the development of hypertension involve, at least in part, abnormal electrolyte handling by the kidney and endothelial dysfunction due to oxidative stress [16,17]. OA has been shown to enhance urinary Na^+^ excretion, but the nephron segment involved was not clarified. Natriuresis was attributed largely to an effect on the distal tube [18,19]. Hence, we evaluated the effects of OA, Me-OA and Br-OA on fluid and electrolyte handling by the kidney and tested the role of proximal tubular Na^+^ reabsorption. In addition we investigated the effects of OA on hormone levels and on oxidative stress in the liver, heart and kidney of DSS and SHR rats.

## Materials and Methods

### Drugs and chemicals

#### Standard chemicals

Except for OA and derivatives which were obtained as described below, drugs were sourced from standard pharmaceutical suppliers. All other chemicals used for this study were of analytical grade and were purchased from standard commercial suppliers.

#### Isolation of OA

**Fig 1:**
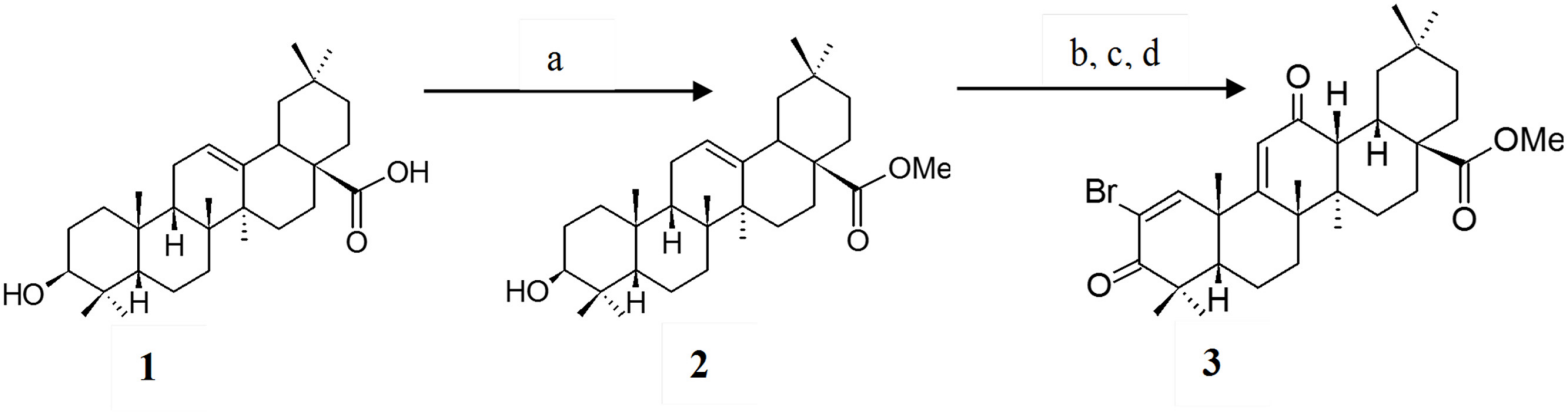
Reaction scheme for the synthesis of OA derivatives as previously described [22]. Reagents: (a) CH_2_N_2_, Et_2_O, THF; (b) IBX, DMSO; (c) *m*CPBA, CH_2_Cl_2_; (d) Br_2_, HBr, AcOH.

OA was isolated from *Syzygium aromaticum* [(Linnaeus) Merrill & Perry] (Myrtaceae) (cloves) using a protocol previously validated in our laboratory with minor modifications [7,10,20]. Briefly, air-dried *S*. *aromaticum* flower buds (500 g) were exhaustively extracted sequentially with dichloromethane (DCM) and ethyl acetate (EA) (twice with 1 L for 24 h for each solvent) at room temperature. The plant material was removed by filtration and the resultant filtrates were concentrated *in vacuo* at 60 ± 1°C using a Büchi rotary evaporator (Buchi Labortechnik AG, Flawil, Switzerland) to obtain DCM soluble (63 g) and ethyl acetate soluble (EAS, 85 g) crude extracts. Crude EAS have been previously shown to contain OA, ursolic acid, methyl maslinate and methyl corosolate [21]. Hence subjecting EAS to column chromatography and recrystallisation from methanol yielded pure OA whose structure was confirmed by spectroscopic analysis using ^1^H and ^13^C nuclear magnetic resonance (NMR). Spectroscopic data indicated that the *S*. *aromaticum*-isolated OA (compound **1** in Fig 1) was pure and similar to commercial OA, hence this triterpene was used for animal studies and as a starting material for the synthesis of oleanane derivatives.

#### Synthesis of Me-OA and Br-OA

OA derivatives were synthesized according to a previously described method [22]. To obtain the methyl ester (Me-OA) (compound **2** in Fig 1), a 40% (m/v) aqueous solution of KOH (7.5 mL, 0.1 mM) was added to diethyl ether (40 mL) followed by addition of 2 g (0.02 M) of nitrosomethylurea at 0°C. The yellow ethereal layer of diazomethane (CH_2_N_2_) was poured into the tetrahydrofuran (THF) (5 mL, 0.07 mM) solution of OA (500 mg, 1.09 mmol). The mixture was left in a fume hood overnight and compound **2** was obtained as a whitish powder (65%) with m.p. 124–126°C. Compound **2** (1.20 g, 2.55 mmol) was then oxidized with iodoxybenzoic acid (IBX; 2.86 g, 10.2 mmol) in dimethyl sulphoxide (DMSO; 35 mL). This was followed by epoxidation of the oxidized product using *m*-chloroperoxybenzoic acid (*m*CPBA; 321 mg, 1.3 mM). The epoxidation product was then brominated with hydrobromic acid (44 μL, 0.38 mM) and bromine (0.12 mL, 1.04 mmol) in acetic acid (10 mL) to yield compound **3** (Fig 1) (30%). An analytically pure sample of this compound was obtained by column chromatography (hexanes-EA, 4:1 to 2:1) as a yellowish solid with m.p. 137–140°C. All structures of synthetic products were confirmed by ^1^H, ^13^C NMR and infrared spectroscopy. Spectra were recorded on a Bruker DRX-400 NMR and a Bruker Alpha FT-IR spectrometer. The pure compounds were used for animal studies.

### Animals

Normotensive (normal) male Wistar (250–300 g), weanling Dahl salt-sensitive (DSS, 100–150 g) and spontaneously hypertensive rats (SHR, 100–150 g) rats bred and housed at Biomedical Research Unit, University of KwaZulu-Natal, were used in this study. The rats were maintained on a 12 h light / dark regime, and given both food (Meadow Feeds, Pietermaritzburg, South Africa), and water *ad libitum*. All experimental protocols were reviewed and approved by the animal ethics committees of the University of KwaZulu-Natal (077/12/Animal; 028/13/Animal) and the University of Leuven (4500768204).

The animals used for chronic studies were given enough time to acclimatise in metabolic cages before the study was undertaken. Animal cages were cleaned out daily. Animals were monitored for pain, discomfort and distress using the criteria listed in the humane end points documents of ethics committee of the University of KwaZulu-Natal and of the University of Leuven.

At the end of the experiments animal carcasses and tissues were packaged in boxes with biohazard symbol approved by the Biological Safety Office for the Safety’s Disposal Service, and transported to the Biomedical Research Animal Unit for disposal.

#### Experimental design

The animals were used to study either 1) acute (4 hours) effects of OA and its oleanane derivatives on MAP and renal function under anaesthesia in normotensive rats, or 2) sub-chronic (9 weeks) effects of OA on MAP, renal function and oxidative stress in normotensive, DSS and SHR rats.

#### Acute studies

Male Wistar rats were fed standard rodent chow supplemented with lithium chloride (12 mmol.kg^-1^ dry weight) for 48 h prior to experimentation in order to raise plasma lithium to measurable concentrations without affecting renal Na^+^ or water excretion. The rats were prepared for acute mean arterial blood pressure and renal function measurements using a protocol previously reported from our laboratories [23]. Briefly, the rats were anaesthetized by intra-peritoneal injection of inactin [5-ethyl-5-(10-methylpropyl)-2 thiobarbiturate, 0.11 g kg^-1^] (Sigma Aldrich, St. Louis, MO, USA) and tracheotomy was performed. The depth of anaesthesia was monitored throughout the experiment and additional intravenous bolus doses of inactin (0.05 g kg^-1^) were administered when necessary. A catheter was implanted in the left carotid artery for withdrawal of blood samples and continuous recording of arterial blood pressure at 30 min intervals via a pressure transducer (Statham MLT 0380, Ad Instruments, Bella Vista, NSW, Australia), compatible with PowerLab System ML410/W (Bella Vista, NSW, Australia). Another catheter was inserted in the right jugular vein for continuous intravenous infusion (Harvard Syringe Infusion Pump 22, Harvard Apparatus, Holliston, MA, USA) of 0.077 M NaCl containing creatinine (0.15 mg mL^-1^) at 9 mL h^-1^ to allow calculation of creatinine clearance as a measure of GFR. A minimal abdominal incision was made and a urinary bladder catheter was inserted for the collection of urine samples. After a 3.5 h equilibration period, blood pressure recordings and urine samples were taken every 30 min and blood samples (200 μL) were drawn hourly for measurements of electrolyte concentrations over a 4 h experiment divided into 1 h control, 1.5 h treatment and 1.5 h recovery periods. Handling of Na^+^ in the proximal tubule was evaluated through measurement of lithium clearance (C_Li_) [24]. In those animals in which the effects of OA or derivatives were examined, the infusate was changed during the 1.5 h treatment period to the one identical in ionic composition, but containing OA, Me-OA and Br-OA (90 μg h^-1^). The infusate was then switched back to the vehicle for a further 1.5 h recovery period. The reabsorption of Na^+^ in the proximal tubule and, by implication, in the distal nephron was assessed through measurement of lithium clearance (C_Li_) [24].

#### Sub-chronic studies

Separate groups of male Wistar, SHR and DSS rats (n = 6 in each group) housed individually in Makrolon polycarbonate metabolic cages (Techniplast, Labotec, South Africa) were treated with various doses of OA (30, 60 and 120 mg kg^-1^, p.o.) twice (at 09h00 and 15h00) every third day for 9 weeks. The rats were given both food and water *ad libitum*. The selection of these doses was based on the posology from previous studies in our standard laboratory [7,9,10,20,21]. OA was freshly dissolved in dimethyl sulphoxide (DMSO, 2 mL) and normal saline (19 mL) [21] before use in each case. Rats given DMSO/saline (3 mL kg^-1^, p.o.) acted as untreated controls. The amounts DMSO used for dissolving OA and related oleanane derivatives for acute (4 hours) and sub-chronic (9 weeks) experiments were selected based on our preliminary studies. Urine volume, and urinary excretion rates of creatinine, urea, Na^+^, K^+^ and Cl^-^ were determined daily (see Biochemical Measurements below) while mean arterial blood pressure (MAP) was monitored every third consecutive day using non-invasive tail cuff method with photoelectric sensors (IITC Model 31 Computerised Blood Pressure Monitor, Life Sciences, Woodland Hills, California, USA) as previously described [4,5]. The equipment was calibrated each day prior to measurements. The animals were kept warm at ± 30°C in an enclosed chamber (IITC Model 303sc Animal Test Chamber, IITC Life Sciences, Woodland Hills, California, USA) for 30 minutes before blood pressure recording. All measurements were conducted at 09h00. Blood samples were collected by cardiac puncture into individual pre-cooled heparinized containers at the end of the 9 week experimental period for biochemical analysis.

### Laboratory measurements

#### Urinalysis

Urine volume was determined gravimetrically using a balance (Mettler balance PC 180-instruments, Protea Laboratory Services, Johannesburg, South Africa). Quantitative measurements of total urinary outputs and plasma concentrations of Na^+^, K^+^, Cl^-^, urea and creatinine were performed using Beckman Coulter (Synchron LX20 Clinical Systems, Fullerton, California, USA) with reagent kits from Beckman Coulter (Synchron LX20 Clinical Systems, Dublin, Ireland). Lithium was determined by flame emission spectroscopy at 670.8 nm (Optima 2100 DV, Perkin Elmer, Shelton, CT) using a modified procedure that has been previously described [7]. Fractional excretion rates of Na^+^ (FE_Na_) and Li^+^ (FE_Li_) were determined simultaneously.

### Plasma aldosterone and arginine vasopressin

#### Aldosterone

A standard enzymatic method was used to determine plasma aldosterone concentrations from blood samples collected from treated and untreated groups of Wistar, SHR and DSS rats using an aldosterone ELISA Kit (DRG International, Springfield, New Jersey, USA). The lower and upper limits of detection were 4 pmol L^-1^–789 pmol L^-1^, respectively. The intra assay analytical coefficient of variation ranged from 9.9 to 12.6% and the inter-assay coefficient variation from 6.0 to 8.5%.

#### Arginine vasopressin (AVP)

Plasma AVP concentrations were measured from blood samples collected from treated and untreated groups of Wistar, SHR and DSS rats using an Arg^8^-Vasopressin ELISA Kit (Cusabio Biotech Wuhan, Hubei, China). The lower and upper limits of detection were 3 pmol L^-1^–923 pmol L^-1^, respectively. The intra assay analytical coefficient of variation ranged from 5.9 to 10.6% and the inter-assay coefficient variation from 6.0 to 8.5%.

#### Antioxidant evaluation in the liver, heart and kidney tissues

At the end of a 9-week experimental period, we compared levels of MDA, a commonly known marker of oxidative stress, and of antioxidant defense enzymes SOD and GPx in the liver, heart and kidney between untreated normotensive Wistar and untreated or OA-treated hypertensive rats to establish the effects of OA on oxidative status.

#### Tissue sample harvesting

All animals were sacrificed by exposing to halothane for 3 min via a gas anaesthetic chamber (100 mg kg^-1^). Thereafter, the liver, kidney and heart were removed, snap frozen in liquid nitrogen and stored in a BioUltra freezer (Snijers Scientific, Tilburg, Netherlands) at −70°C until use. All organs were analyzed for protein content in addition to other biochemical parameters. The protein content was quantified using the Lowry method [25]. All the samples were standardized to one concentration (1 mg mL^-1^).

#### MDA

MDA levels in heart, kidney and liver tissues from Wistar, DSS and SHR rats were estimated using a method which has been previously described [26]. Tissues (50 mg) were homogenised in 500 μL of 0.2% phosphoric acid. The homogenates were centrifuged at 14,000 x g at 4°C for 10 minutes. Thereafter, 400 μL of the homogenate was supplemented with 400 μL 2% phosphoric acid and then separated into two glass tubes, each receiving equal volumes of the solution. Subsequently, 200 μL of 7% phosphoric acid was added into both glass tubes followed by the addition of 400 μL of TBA/butylated hydroxytoluene (BHT) into one glass tube (sample test) and 400 μL of 3 mM hydrochloric acid (HCl) into the second glass tube (blank). To ensure an acidic pH of 1.5, 200 μL of 1 M HCl was added to sample and blank test tubes. Both solutions were heated at 100°C for 15 min, and allowed to cool to room temperature. Butanol (1.5 mL) was added to the cooled solution; the sample was vortexed for 1 min to ensure rigorous mixing and allowed to settle until 2 phases could be distinguished. The butanol phase (top layer) was transferred to Eppendorf tubes and centrifuged at 13200 x g for 6 min. The samples were aliquoted into a 96-well microtitre plate in triplicate and the absorbance was read at 532 nm (reference λ 600 nm) on a BioTek μQuant spectrophotometer (Biotek, Johannesburg, South Africa). The absorbance from these wavelengths was used to calculate the concentration of MDA using Beer’s Law. The homogenized tissue concentration of MDA was calculated using the equation below and expressed as units per gram protein.

$$\left[MDA\right]\;\left({nmol\;g}^{-1}\right)\;=\;\frac{Average\;absorbance}{{Absorption\;coefficient\;(156\;mM}^{-1})}$$

#### SOD

Tissue SOD activities from homogenates of heart, kidney and liver of Wistar untreated and treated, DSS and SHR rats were assessed by measuring the dismutation of superoxide radicals generated by xanthine oxidase and hypoxanthine in a convenient 96 well format using a commercially available BioVision SOD Assay Kit (BioVision Research Products, Mountain View, California, USA). Xanthine and hypoxanthine oxidase were used to generate superoxide radicals, which react with 2-(4-iodophenyl)-3-(4-nitrophenol)-5-phenyltetrazolium chloride (INT) to form a red formazan dye. SOD activity was then measured by the degree of inhibition of this reaction. The homogenized tissue activity of SOD was calculated using the equation below and expressed as units per gram protein.

$$SOD\;activity\;\left({nmol\;min}^{-1}{\;mL}^{-1}\right)\;=\;\frac{\left(A\;blank\;1-A\;blank\;3\right)\;-\;(A\;sample\;1-\;A\;blank\;2)}{\left(A\;blank\;1-A\;blank\;3\right)}\;\times 100$$

#### GPx

GPx activities were measured in untreated and treated liver, kidney and heart tissues of Wistar, DSS and SHR rats using the Biovision GPx Assay Kit according to manufacturers’ instructions (BioVision Research Products, Mountain View, California, USA). The tissues (50 mg) were homogenized on ice in cold assay buffer (0.2 mL) and subsequently centrifuged at 10000 x g for 15 min at 4°C. GPx catalyzes the oxidation of glutathione by cumene hydroperoxide. In the presence of glutathione reductase and NADPH, the oxidized glutathione is immediately converted to its reduced form with a concomitant oxidation of NADPH to NADP^+^. The decrease in absorbance at 340 nm related to GPx activity is calculated using the equation below, expressed as units per gram protein.

$$GPx\;activity\;\left({nmol\;min}^{-1}{mL}^{-1}\right)\;=\;\frac{\left(B\;-\;B^{0}\right)}{\left(T2\;-\;T1\right)\;\times \;V}\;\times sample\;dilution$$

#### Data management and statistical analysis

All data were expressed as means ± standard error of means (SEM). Glomerular filtration rate, as assessed by creatinine clearance (C_Cr_) was calculated using the standard formulae from measurements of the plasma and urinary concentrations of creatinine and urine flow rate. Renal clearances (C) and fractional excretions (FE) were calculated with the standard formulae where C = U *x* V/P, FE_Li_ = 100 *x* C/GFR; (FE_Li_ = FE_Na prox_), and FE_Na distal_ = C_Na_/C_Li_, where U is the urinary concentration, V is the urine flow rate and P is the plasma concentration.

Data were analyzed using GraphPad InStat Software (version 5.00, GraphPad Software, San Diego, CA, USA). Statistical comparison of the differences between the control and experimental groups was performed with one-way analysis of variance, followed by Tukey—Kramer multiple comparison test. Correlation between changes in MAP and Na^+^ excretion was analyzed using Origin (Originlab, Northampton, MA, USA). When comparing data a difference with p < 0.05 was considered significant.

## Results

### Structures of OA, Me-OA and Br-OA

The purity of the plant-derived OA was approximately 98% and the percentage yield obtained from the plant-material varied from 0.79% to 1.72%. The percentage yield of the synthetic derivatives, Me-OA and Br-OA was 65% and 30%, respectively. The structural data from ^1^H, ^13^C-NMR and IR spectroscopic analysis of the compounds obtained after recrystallization of the compounds with methanol were comparable with literature data [11,22,27,28].

**OA [C_30_H_47_O_3_]**. ^**1**^ **H NMR (400 MHz, CDCl**_**3**_ **)**: δ 5.30 (t, 1H, *J* = 3.5 Hz), 3.21 (dd, 1H, *J* = 11.5 and 4.4 Hz), 2.85 (dd, 1H, *J* = 13.8 and 4.3 Hz), 1.21 (s, 3H), 0.99 (s, 3H), 0.92 (s, 3H), 0.91 (s, 3H), 0.90 (s, 3H), 0.79 (s, 3H), 0.72 (s, 3H).

^**13**^ **C NMR (400 MHz, CDCl**_**3**_ **)**: δ 183.5, 143.8, 122.8, 79.2, 55.4, 47.8, 46.7, 46.1, 41.8, 41.2, 39.4, 38.9, 38.6, 37.3, 34.0, 33.2, 32.8, 32.6, 31.8, 28.3, 27.9, 27.3, 26.1, 23.6, 23.1, 22.9, 18.5, 17.3, 15.7, 15.5.

**IR**: 3454, 2940, 2860, 1688, 1462, 1387, 1274, 1185, 1030,781, 655 cm^-1^.

**Me-OA [C_31_H_50_O_3_]**. ^**1**^ **H NMR (400 MHz, CDCl**_**3**_ **)**: δ 5.30 (t, 1H, *J* = 3.5 Hz), 3.63 (s, 3H), 3.21 (dd, 1H, *J* = 11.5 and 4.4 Hz), 2.85 (dd, 1H, *J* = 13.8 and 4.3 Hz), 0.99 (s, 3H), 0.92 (s, 3H), 0.91 (s, 3H), 0.90 (s, 3H), 0.79 (s, 3H), 0.72 (s, 3H).

^**13**^ **C NMR (400 MHz, CDCl**_**3**_ **)**: δ 178.1, 143.9, 122.6, 79.2, 55.2, 51.5, 47.6, 46.7, 45.9, 41.7, 41.3, 39.3, 38.7, 38.4, 37.0, 34.0, 33.1, 32.7, 32.4, 30.7, 28.1, 27.7, 27.2, 26.0, 23.6, 23.1, 18.3, 16.8, 15.6, 15.2.

**IR**: 3352, 2941, 2860, 1725, 1711, 1462, 1385, 1361, 1163, 1030, 780, 752 cm^-1^.

**Br-OA [C_31_H_43_BrO_4_]**. ^**1**^ **H NMR (400 MHz, CDCl**_**3**_ **)**: δ 7.82 (s, 1H), 5.97 (s, 1H), 3.69 (s, 3H), 3.02 (d, 1H, *J* = 13.7 Hz), 2.91 (d, 1H, *J* = 4.6 Hz), 1.44 (s, 3H), 1.29 (s, 3H), 1.23 (s, 3H), 1.17 (s, 3H), 1.00 (s, 3H), 0.99 (s, 3H), 0.87 (s, 3H)

^**13**^ **C NMR (400 MHz, CDCl**_**3**_ **)**: δ 178.3, 170.2, 155.1, 123.7, 122.1, 67.9, 51.8, 49.6, 48.1, 47.3, 46.1, 45.7, 44.7, 42.1, 35.8, 34.7, 34.4, 33.2, 32.7, 31.8, 31.5, 30.6, 28.0, 27.7, 27.0, 24.6, 23.1, 22.7, 22.0, 21.6, 20.6. **IR**: 2950, 2870, 2253, 1720, 1685, 1660, 1458, 1385, 915, 744, 651, 623, 456 cm^-1^.

### Acute effects of OA, Me-OA and Br-OA

#### GFR and MAP measurements

**Fig 2:**
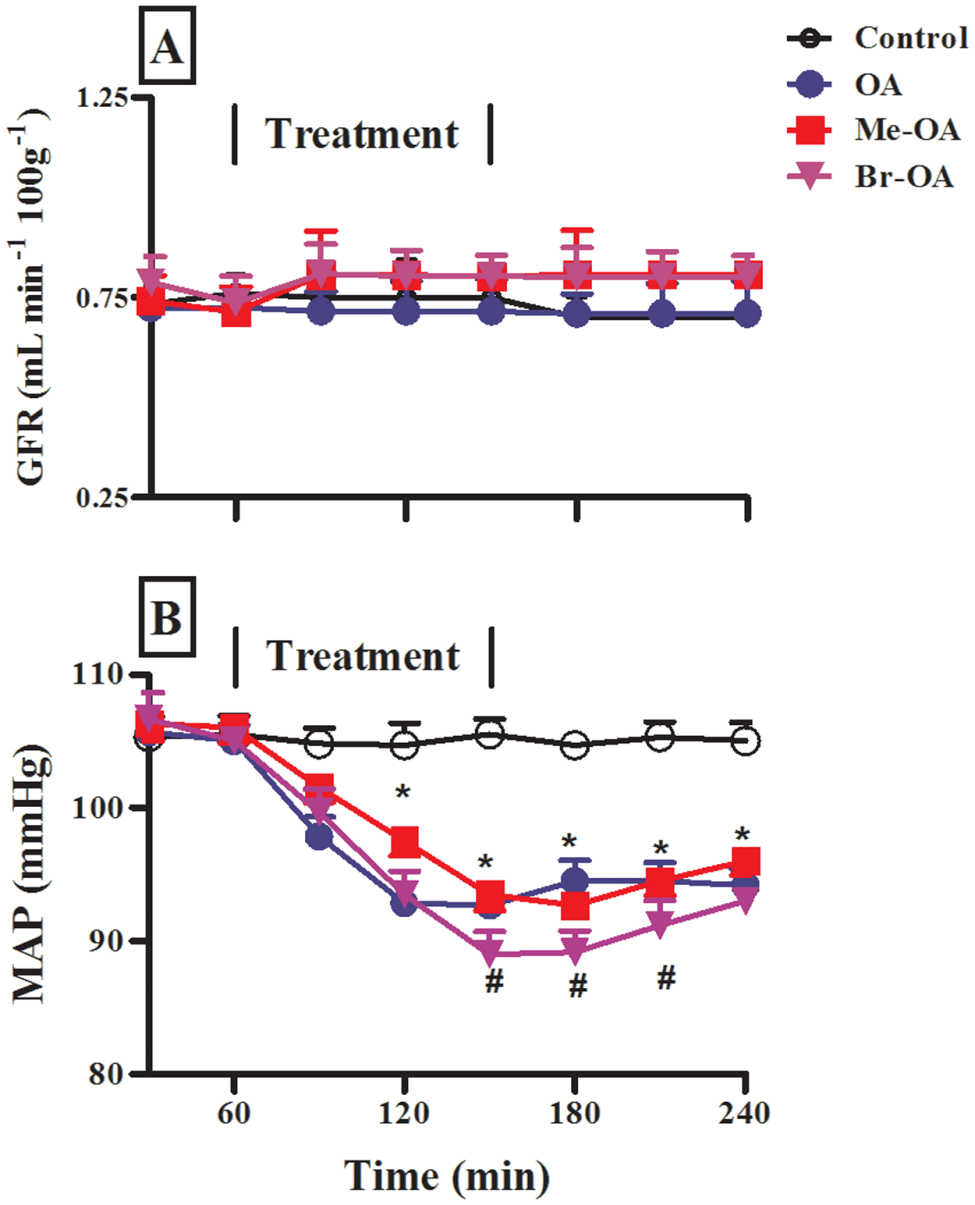
Comparison of GFR (A) and MAP (B) of control (untreated) rats and animals administered OA, Me-OA and Br-OA (90 μg h^-1^) during the 4 h experimental period. All drugs were administered for 1.5 h during the treatment period. Values are presented as means, and vertical bars indicate SEM (n = 6 in each group). * p < 0.001 by comparison with control animals at each corresponding time. # p < 0.05 by comparison with OA-treated animals.

Following infusion of hypotonic saline to control rats, no significant variations were seen in the GFR and MAP throughout the 4 h post-equilibration period (Fig 2). In addition, intravenous infusion of OA or derivatives did not significantly change GFR. On the other hand, intravenous infusion of OA (90 μg h^-1^) for 1.5 h in the experimental group significantly (p < 0.001) reduced MAP by comparison with the control group (n = 6). Similarly, Me-OA and Br-OA decreased MAP, with the hypotensive effects of Br-OA being significantly (p<0.05) more pronounced than those of OA. The MAP of drug infused rats did not revert back to pre-treatment levels during the recovery period.

#### Fluid and electrolyte handling

**Fig 3:**
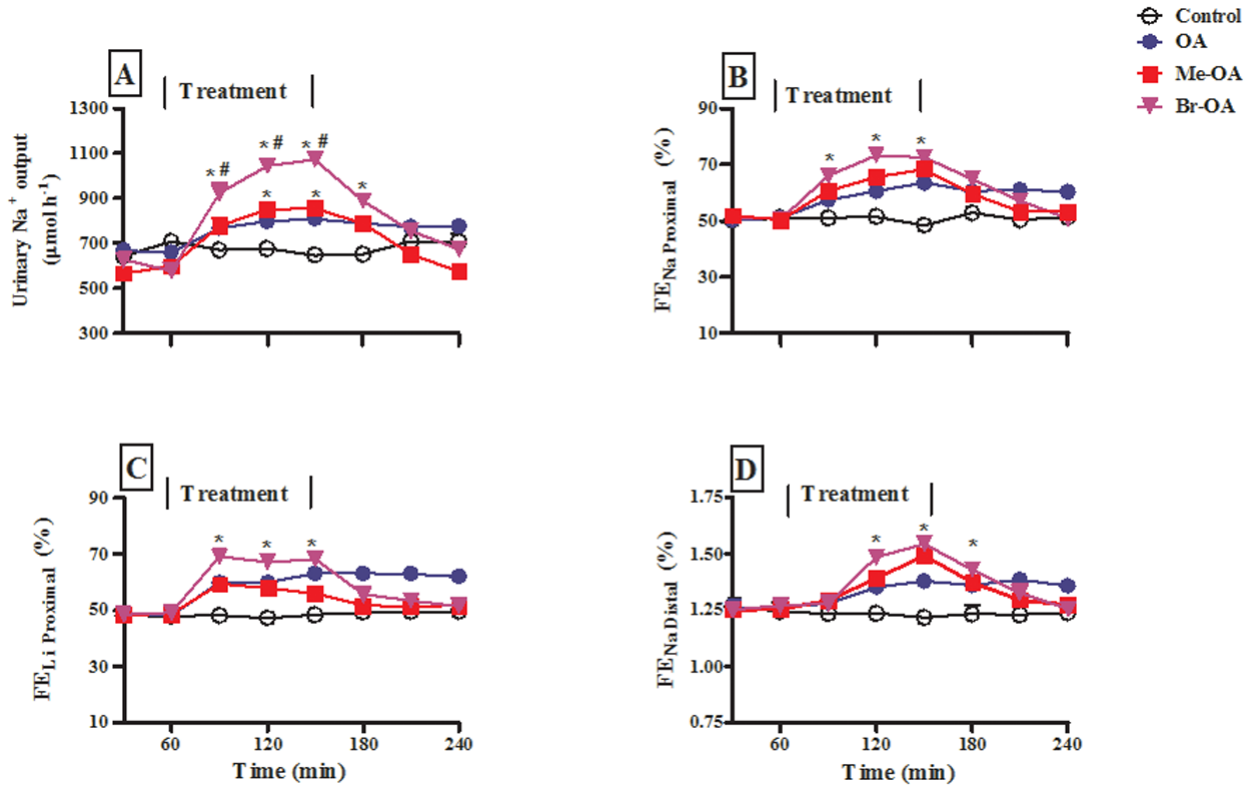
Comparison of (A) urinary Na^+^ excretion (B), FE_Na proximal_ tubule (C) FE_Li proximal_, and FE_Li distal_ (D) of control rats and animals infused OA, Me-OA and Br-OA. Drugs were administered for 1.5 h during the treatment period. Values are presented as means, and vertical bars indicate SEM (n = 6 in each group). * p < 0.05 by comparison with control animals at each corresponding time. # p < 0.05 by comparison with OA-treated animals.

Urine flow rate under control conditions (i.e. in untreated animals, as well as before treatment in the treated group) matched the infusion rate of 9 mL h^-1^. OA, Me-OA and Br-OA infusion had no influence on urine flow rate (not illustrated, but see Table 1). Fig 3A shows urinary Na^+^ excretion rates in the same animals. Urinary Na^+^ excretion rates of control animals was stable throughout the experiment and was comparable to the infusion rate of 693 μmol h^-1^. OA and the oleanane derivatives Me-OA and Br-OA significantly (p < 0.05) increased urinary Na^+^ excretion rates during the treatment period by comparison with control animals. The effects of Br-OA were significantly (p<0.05) more pronounced than those of OA. This is reflected in Table 1 which shows calculated cumulative data for urine flow and cumulative amounts of Na^+^ (and other electrolytes) excreted during the 1.5 h treatment period.

**Table 1:** Comparison of the effects of OA and derivatives infusion on urine flow and total amount of electrolytes excreted during 1.5 h treatment period.

	Parameter			
Group	Urine volume (mL)	Na^+^ (mmol)	K^+^ (mmol)	Cl^-^ (mmol)
Control	14.00 ± 0.16	0.98 ± 0.02	0.24 ± 0.02	1.04 ± 0.01
OA	14.00 ± 0.15	1.16 ± 0.01 *	0.24 ± 0.02	1.08 ± 0.04
Me-OA	14.02 ± 0.07	1.24 ± 0.03 *	0.22 ± 0.01	1.24 ± 0.01 *
Br-OA	13.66 ± 0.07	1.52 ± 0.04 * #	0.20 ± 0.03	1.30 ± 0.03 * #

* p < 0.05 by comparison with control animals

# p < 0.05 by comparison with OA-treated animals.

Values are presented as means ± SEM (n = 6 in each group).

The effects of intravenous infusion of OA and derivatives on Na^+^ reabsorption in the proximal tubule and, by implication, in the distal nephron was assessed through measurement of lithium clearance (C_Li_), in anaesthetized Wistar rats supplemented with LiCl 48 h prior to experimentation. The plasma Li^+^ concentrations of animals which varied between 0.2 and 0.3 mmol L^-1^ showed no statistical difference between the groups. Fractional excretions (FE) rates of Na^+^ (FE_Na_) and Li^+^ (FE_Li_) (an index of end-proximal FE_Na_ delivery) [24] were determined simultaneously. Prior to the infusion of OA and related oleanane derivatives, the FE_Li_ and FE_Na_ of anaesthetized saline infused control animals and experimental groups did not differ between groups (Fig 3B and 3C). Infusion of OA and related oleanane derivatives over the 1.5 h treatment period caused substantial increases in FE_Li_ and FE_Na_ by comparison with control animals at the corresponding time periods (Fig 3B and 3C). Finally, FE_Na dist_ (C_Na_/C_Li_), used as an index of the fraction of sodium delivered to the distal nephron that escapes reabsorption therein, was also significantly elevated (Fig 3D). In all cases, the FE_Na_ was not accompanied by any changes in FE_K_, (data not shown).

**Fig 4:**
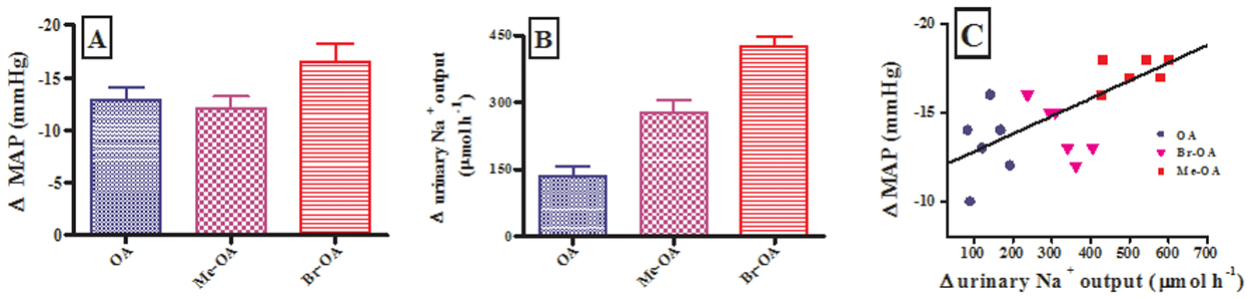
Comparison of the changes in MAP (A), urinary Na^+^ excretion (B); correlation between MAP and Na^+^ changes (C) in OA or derivative-administered animals during the 1.5 h treatment period. In panels A and B, group values are presented as means, and vertical bars indicate SEM (n = 6 in each group). In panel C, individual values of each animal are presented.

Fig 4A and 4B show the group changes in MAP and Na^+^ excretion rates, respectively, at the end of the treatment period, and Fig 4C shows the relationship between these changes as analyzed by individual values of each animal from the control and experimental groups. Increased urinary Na^+^ excretion rate in these acute experiments had some but weak correlation with decreased MAP (R = 0.67).

In summary, acute infusion of OA or related synthetic derivatives increased urinary Na^+^ excretion rates and FE_Li_ without influencing GFR indicating that at least part of the overall increase in natriuresis was due to a reduction in Na^+^ reabsorption in the proximal tubules.

### Sub-chronic effects of OA

The above results indicate that OA and related derivatives have marked effects on the Na^+^ handling by the kidney under acute conditions. To examine whether the effects observed under acute conditions can be preserved or modified with time, we measured the same parameters for a sub-chronic period of 9 weeks in conscious Wistar, SHR and DSS rats. These experiments were restricted to OA because of the poor yield of synthetic derivatives obtained during the phytochemical studies.

#### Arterial pressure

**Fig 5:**
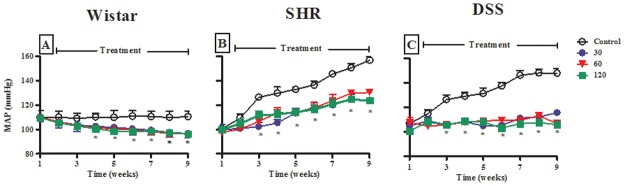
Effects of the administration of various doses of OA (30, 60, 120 mg kg^-1^) on MAP Wistar (A), SHR (B) and DSS (C) rats over a 9-week experimental period. Values are presented as means, and vertical bars indicate SEM (n = 6 in each group). * p < 0.05 by comparison with control animals at each corresponding time (shown only for 120 mg kg^-1^ for image clarity).

Weekly MAP of control Wistar animals was stable around 110 ± 2 mmHg throughout the 9-week experimental period (Fig 5A, unfilled symbols). Administration of various doses of OA (30, 60, 120 mg kg^-1^ p.o.) significantly reduced MAP (p < 0.05) from the 3^rd^ week until the end of the study period (Fig 5A, filled symbols). In contrast to control Wistar animals, weekly MAP of untreated SHR and DSS animals progressively increased to values > 155 ± 2 mmHg by the end of the experiment (Fig 5B and 5C, unfilled symbols). OA administration in SHR and DSS significantly (p < 0.05) reduced MAP (Fig 5, filled symbols), with more marked effect in DSS rats Comparison of the blood lowering effects of the 3 doses within each animal group did not show any dose dependence.

#### Fluid and electrolyte handling

**Fig 6:**
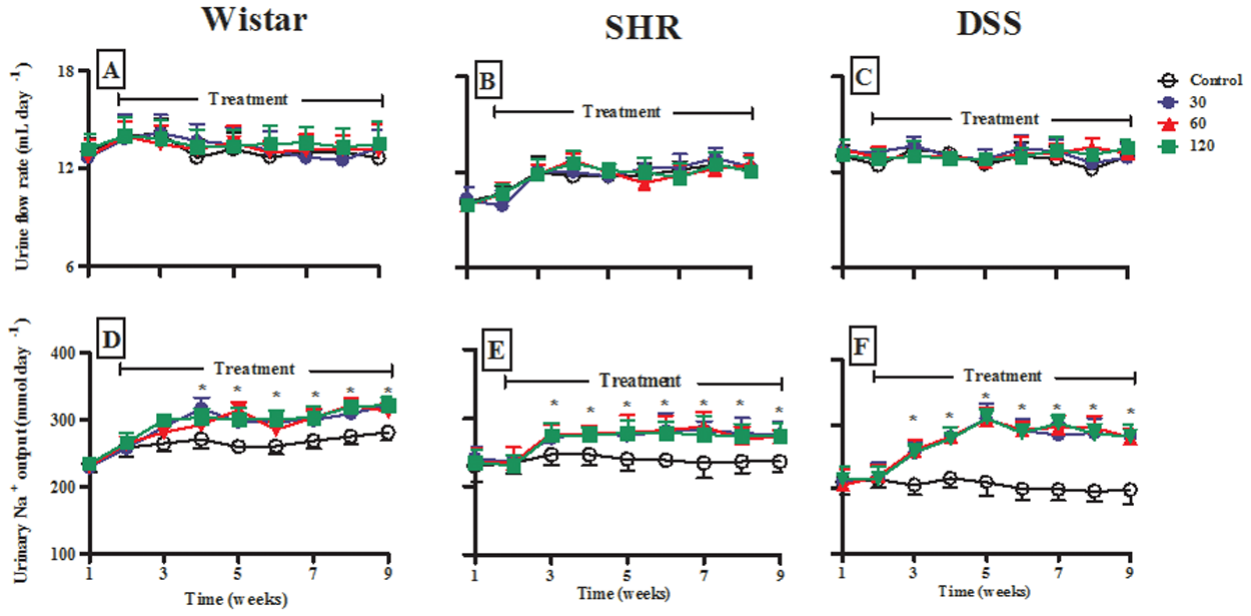
Effects of the administration of various doses of OA (30, 60, 120 mg kg^-1^) on 24 h urine flow (A-C) and Na^+^ excretion (D-F) rates with control Wistar, SHR and DSS animals over a 9-week experimental period. Values are presented as means, and vertical bars indicate SEM (n = 6 in each group). * p < 0.05 by comparison with control animals at each corresponding time.

Fig 6A–6C show the urine flow rate of control and OA-administered Wistar, SHR and DSS animals during the 9-week experimental period. OA administration had no significant influence on the urine flow rate. Fig 6D–6F shows urinary Na^+^ excretion rates in the same animals. Weekly urinary Na^+^ excretion rates of control Wistar animals seemed to spontaneously increase with time without reaching statistical significance by the end of the experimental period. However, in both hypertensive models no such increase with time was obtained, instead, urinary Na^+^ excretion rates tended to decrease with time after weanling in the DSS model. OA had no significant influence on water consumption (29 ± 1 vs 30 ± 2; 29 ± 1 vs 31 ± 2 and 40 ± 1 vs 37 ± 2 mL day^-1^; untreated vs treated with 120 mg kg^-1^ OA in non-hypertensive, SHR and DSS, respectively). Similarly, OA administration had no significant influence on body weight changes throughout the experimental period (increase after 9 weeks: 15.6±0.4%, 16.4±0.8%, and 16.0±0.4% with 120 μg/kg, in Wistar, SHR and DSS groups, respectively; n = 6 in each group) when compared with respective untreated animals (increase after 9 weeks: 14.8±0.8%, 16.0±0.8%, 15.2±0.4% in untreated Wistar, SHR and DSS rats, respectively; n = 6).

**Fig 7:**
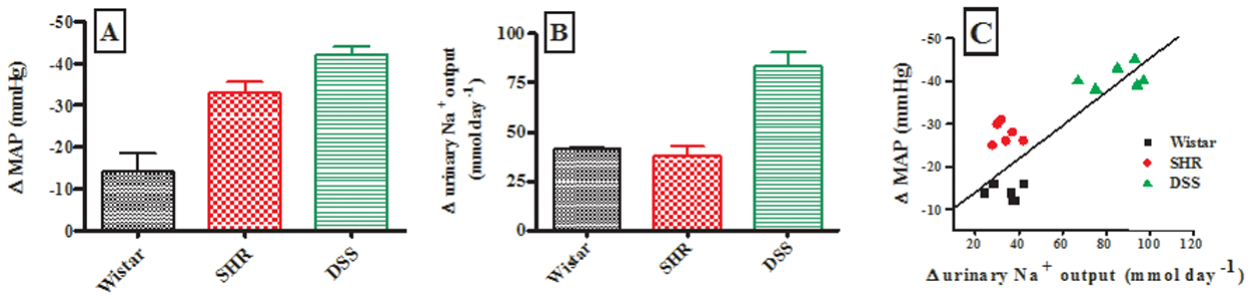
Comparison of the effects of the administration of various doses of OA (30, 60, 120 mg kg^-1^) on changes in MAP (A) and urinary Na^+^ excretion rate (B) on the 9^th^ week of the study. Correlation of MAP and Na^+^ (C) changes in conscious Wistar, SHR and DSS rats. In panels A and B, group values are presented as means for weekly measurements; vertical bars indicate SEM of means (n = 6) in each group. In panel C, individual values of each animal are presented.

Administration of various doses of OA (30, 60, 120 mg kg^-1^ p.o.) significantly (p < 0.05) increased weekly urinary Na^+^ excretion rates in Wistar, SHR and DSS rats from the 3^rd^ week until the end of the study period (Fig 6D–6F). The increase was most marked in DSS rats (Fig 6F, see also Fig 7B). We analyzed the relationship between OA-induced changes in urinary Na^+^ excretion rates and MAP changes in individual animals on the last week of the study. We observed a linear relationship (R = 0.83, p < 0.0001) between increase in urinary Na^+^ excretion rate and decrease in MAP (Fig 7C). The OA-evoked increase in urinary Na^+^ excretion rates had no impact on plasma Na^+^ concentration measured at the end of the experiment in Wistar and SHR rats, but there was a baseline relative hypernatremia in DSS rats, which was resolved after treatment (Table 2). In addition, there was a significant (p < 0.05) increase in GFR with a concomitant decrease in plasma creatinine concentration at the end of the experiment in OA-treated Wistar, SHR and DSS rats (Table 2).

**Table 2:** The effects of OA on plasma biochemical parameters in male Wistar, SHR and DSS rats which were administered OA twice every third day for nine weeks.

		Experimental Protocol			
Parameter	Group	Control	OA30	OA 60	OA 120
Na^+^ (mmol L^-1^)	Wistar	142 ± 1	142 ± 2	141 ± 2	140 ± 1
	SHR	142 ± 2	137 ± 2	138 ± 2	140 ± 1
	DSS	157 ± 1*	137 ± 1	139 ± 2	137 ± 3
K^+^ (mmol L^-1^)	Wistar	4.3 ± 0.2	4.1 ± 0.2	4.0 ± 0.2	4.2 ± 0.2
	SHR	4.8 ± 0.1	5.1 ± 0.2	4.9 ± 0.2	5.2 ± 0.2
	DSS	5.4 ± 0.1	6.3 ± 0.1	6.2 ± 0.3	6.2 ± 0.5
Cl^-^ (mmol L^-1^)	Wistar	105 ± 2	103 ± 2	106 ± 1	102 ± 2
	SHR	100 ± 3	109 ± 6	102 ± 3	104 ± 4
	DSS	105 ± 2	103 ± 4	102 ± 1	102 ± 5
Urea (mmol L^-1^)	Wistar	7.8 ± 0.6	8.2 ± 0.6	8.4 ± 0.5	8.5 ± 0.3
	SHR	7.0 ± 0.2	7.2 ± 0.4	7.4 ± 0.4	6.7 ± 0.4
	DSS	8.7 ± 0.4	9.1 ± 0.3	8.3 ± 0.5	7.9 ± 0.3
Creatinine (μmol L^-1^)	Wistar	28 ±2	21 ± 1*	20 ± 2*	20 ± 5*
	SHR	34 ±4	27± 5*	26 ± 2*	27 ± 4*
	DSS	33 ±1	27 ± 2*	26 ± 1*	24 ± 2*
GFR (mL min^-1^ 100 g^-1^)	Wistar	0.47 ±0.16	0.68 ±0.18*	0.76 ±0.17*	0.85 ±0.15*
	SHR	0.48 ±0.14	0.74 ±0.16*	0.86 ±0.17*	0.85 ±0.17*
	DSS	0.32 ±0.16	0.98 ±0.18*	0.98 ±0.18*	0.71 ±0.15*
Kidney (g 100 g^-1^)	Wistar	0.45 ± 0.20	0.47 ± 0.05	0.46 ± 0.07	0.47 ± 0.02
	SHR	0.62 ± 0.06	0.51 ± 0.07	0.62 ± 0.08	0.58 ± 0.05
	DSS	0.68 ± 0.02	0.48 ± 0.01	0.57 ± 0.2	0.49 ± 0.02
Heart (g 100 g^-1^)	Wistar	0.06 ± 0.04	0.05 ± 0.03	0.06 ± 0.04	0.07 ± 0.01
	SHR	0.07 ± 0.01	0.09 ± 0.02	0.09 ± 0.01	0.07 ± 0.01
	DSS	0.08 ± 0.05	0.08 ± 0.05	0.09 ± 0.07	0.09 ± 0.10

* p < 0.05 by comparison with respective control animals

Values are presented as means ± SEM (n = 6 in each group).

#### Effects of OA on aldosterone and arginine vasopressin

The effects of OA on aldosterone and AVP secretion are shown in Table 3. Untreated SHR and DSS rats exhibited elevated plasma aldosterone and AVP concentrations compared to Wistar control animals. By comparison with respective control animals, OA administration significantly decreased the plasma aldosterone concentrations (p < 0.05) in the hypertensive rats. OA treatment had no influence on the secretion of AVP in comparison to respective control animals.

**Table 3:** The effects of OA on terminal plasma hormone measurements in male Wistar, SHR and DSS rats which were administered OA twice every third day for nine weeks.

	Group	Aldosterone (nmol L^-1^)	AVP (pmol L^-1^)
**Sub-chronic study**	Wistar control	0.77 ± 0.04	2.28 ± 0.03
	SHR control	1.30 ± 0.25*	2.78 ± 0.08*
	DSS control	1.48 ± 0.48*	2.85 ± 0.07*
	SHR OA	1.02 ± 0.02#	2.61 ± 0.05*
	DSS OA	± 0.02#	2.74 ± 0.06*

* p < 0.05 by comparison with Wistar control animals

# p < 0.05 by comparison with respective control animals.

Values are presented as means ± SEM (n = 6 in each group).

#### Oxidative status in the heart, kidney and liver tissues

Tissues were harvested at the end of the 9-week study and assessed for oxidative status. The concentrations of MDA and antioxidant enzymes (SOD and GPx) in Wistar control animals represent baseline/normal activity levels found in the tissues used. Significant increases of MDA and decreases of SOD and GPx were found in untreated liver, heart and kidney tissues of SHR and DSS rats as compared to control Wistar animals (Table 4). Treatment of SHR and DSS rats with OA (60 mg kg^-1^, p.o.) for 9 weeks significantly reduced the MDA in all tissues and increased the activities of SOD and GPx in the liver and kidney compared to untreated hypertensive animals. There were no differences in magnitude of the effects of the three doses of OA on blood pressure parameters measured above in this study, hence only a median dose treatment was selected for this *in vitro* analysis of oxidative proteins. On the other hand, GPx activity was undetectable (<0.01 nmol.min^-1^ mL^-1^ g protein) in the liver of SHR and DSS rats.

**Table 4:** Comparison of MDA concentration, activities of SOD and GPx in the kidney, heart and liver harvested at the end of the study from SHR and DSS rats treated with OA twice every third day for 9 weeks with control Wistar rats.

		Tissue		
Parameter measured	Experimental protocol	Heart	Kidney	Liver
**MDA (nmol.g** ^**-1**^ **protein)**	Wistar control	2.12 ± 0.09	1.70 ± 0.05	1.52 ± 0.06
	SHR control	2.75 ± 0.01	2.05 ±0.02*	2.46 ±0.04*
	DSS control	5.10 ± 0.10*	4.02 ± 0.08*	4.18 ± 0.09*
	SHR OA	1.16 ±0.01#	1.21 ±0.01#	1.35 ± 0.02#
	DSS OA	1.82 ± 0.20#	1.55 ± 0.10#	1.71 ± 0.30#
**SOD activity (nmol.min** ^**-1**^ **mL** ^**-1**^ **g protein)**	Wistar control	6.11 ± 0.30	10.20 ± 1.04	7.05 ± 0.13
	SHR control	2.13 ± 0.48*	3.01 ± 0.21*	3.56 ± 0.41*
	DSS control	1.25 ± 0.08*	2.29 ± 0.07*	2.12 ± 0.14*
	SHR OA	5.78 ± 0.14#	9.26 ± 0.12#	6.84± 0.07#
	DSS OA	4.12 ± 0.33#	11.08 ± 0.41#	8.72 ± 0.32#
**GPx activity (nmol.min** ^**-1**^ **mL** ^**-1**^ **g protein)**	Wistar control	3.82 ± 0.16	2.38 ± 0.21	0.10±0.02
	SHR control	0.62±0.06*	0.60±0.02*	Undetectable
	DSS control	0.60 ± 0.02*	0.20 ± 0.06*	Undetectable
	SHR OA	3.47±-0.03#	3.08±0.02#	Undetectable
	DSS OA	4.14 ± 0.31#	3.38 ± 0.24#	Undetectable

Note: undetectable ≤ 0.01 nmol.min^-1^ mL^-1^ g protein

* p < 0.05 by comparison with Wistar control animals;

# p < 0.05 by comparison with respective control animals.

Values are expressed as mean ± SEM (n = 6).

## Discussion

The present study investigated the effects of OA and related synthetic derivatives (Me-OA and Br-OA) on blood pressure and thereafter examined the possible underlying mechanisms. Our results show that 1) OA decreased MAP and increased urinary Na^+^ excretion in both acute and sub-chronic experimental settings; 2) OA derivatives had qualitatively similar effects (but quantitatively more marked increase of Na^+^ output with Br-OA) in acute experiments; 3) the MAP lowering effects of OA under sub-chronic settings were more marked in hypertensive animals compared to normotensive rats; 4) the acute natriuretic action was obtained without change in GFR but with an increase of FE_Na_; 5) OA increased GFR under sub-chronic administration, 6) plasma levels of aldosterone were increased in the hypertensive animals and were decreased by OA treatment; 7) there was no drug increased urinary flow rate and no influence on AVP levels in comparison to respective control animals; 8) OA suppressed the increased ROS production present in salt-sensitive hypertensive rats and modulated the production of antioxidant enzymes, SOD and GPx, which were reduced in these animals. We did not observe any increased mortality in the OA-treated animals compared to untreated ones. This result is different from the increased weight loss and mortality observed by Hong *et al*. [29]. We do not have an explanation for the difference, but it could be that rats are more resistant to OA compared to mice.

The other aim of the study was to test whether two synthetic derivatives of OA display effects similar to those of the parent drug. Our data in the acute experimental setting clearly demonstrate similar hypotensive and increased urinary Na^+^ output effects. Due to limitation of the availability of the derivatives only OA was used in chronic studies in experimental hypertensive animals. SHR and DSS rats are well studied animal models of essential (primary) arterial hypertension, and both were used together with a group of normotensive Wistar controls in our experimental setting involving 9 weeks. Our data confirm that blood pressure, indeed increase progressively with time in the absence of drug treatment in hypertensive animals whereas it remained stable in the control animals (see Fig 5). OA treatment significantly suppressed or blunted this increase, but also reduced blood pressure in control animals. Our results confirm the previously reported antihypertensive properties of OA in experimental models of hypertension [7,19]. The present study demonstrates for the first time a more marked action in SHR and DSS by comparison with non-hypertensive animals, suggesting a specifically enhanced action in disease conditions. The pathophysiological mechanisms responsible for the rise in blood pressure in primary hypertension remain unclear and are likely complex. Amongst the mechanisms accepted to play a major role for the development of hypertension is an inability of the kidney to excrete Na^+^; indeed solute retention and the accompanying water retention result in extracellular (including intravascular) volume expansion [18,30]. Interestingly, the hypotensive and urinary Na^+^ excretion effects of various doses of OA (30, 60, 120 mg kg^-1^ p.o.) were not concentration-dependent which corroborates our previous observations with similar dosages. We attribute this to the narrow range of the doses used [7]. Further studies with a wider range of OA doses are expected to provide this information. We were therefore interested in examining whether the marked hypotensive action obtained in the hypertensive models could be correlated with an effect on Na^+^ handling by the kidney. We noticed that urine Na^+^ excretion rate in normotensive control, untreated animals tended to spontaneously increase with time during the following 9 weeks post weanling. In both hypertensive models, no such increase with time was obtained, instead, urinary Na^+^ excretion rate tended to decrease with time after weanling in rats consistent with Na^+^ retention previously reported in these animals [18,30].

To further support this hypothesis of Na^+^ retention we found increased aldosterone levels in plasma of non-treated hypertensive rats. Our data show that OA decreased aldosterone secretion and increased urine Na^+^ excretion rate, with significantly larger increases in Na^+^ output being obtained in the DSS hypertensive models. This suggests that treatment with the drugs was accompanied by alleviation of Na^+^ retention in these animals. Our data demonstrate that the natriuretic effects of OA and related derivatives was associated with increased Li^+^ clearance. This indicates that the increase in urinary Na^+^ excretion rate is, at least in part, mediated via inhibition of proximal tubular Na^+^ reabsorption. The results also indicate that OA and related derivatives increased FE_Na dist_ partly as an inherent response of reduced proximal tubular reabsorption. It is likely that there is also a contribution of decreased distal tubular reabsorption as a consequence of the decrease in plasma aldosterone concentration. Although the effects of OA and related derivatives on renal fluid, Na^+^, K^+^ and Cl^-^ have been studied, renal handling of other ions such as Mg^2+^, Ca^2+^ and PO_4_^2-^ warrant investigations.

We found a positive correlation between the increase in urinary Na^+^ excretion rate and the decrease in MAP (see Figs 4 and 7). However, despite the potent natriuretic effects of these triterpenes, the urine flow rate was not changed as supported by unchanged levels of AVP by comparison with respective control animals. OA and derivative-evoked blood pressure lowering effects in the absence of increased fluid voided in the urine and effects on water consumption indicate that there was no change in volume expansion and may imply an effect on other, non-renal mechanisms. OA has been shown to induce vasorelaxation of aortic rings from normal and hypertensive animals, as well as relaxation in mesenteric arteries [29,31,32]. Vasodilatation and the resulting decrease of total peripheral resistance could be implicated in the MAP lowering effect obtained in our experiments, but this mechanism was not explored in the present study. The improved Na^+^ elimination (decreased Na^+^ retention) caused by drug treatment, although not resulting in changes of extracellular volume, could still have an indirect effect on vascular function. Decrease in extracellular Na^+^ concentration can affect Na^+^ concentration gradients and influence the activity of the Na^+^-Ca^2+^ exchanger, resulting in changed Ca^2+^ efflux or entry via this pathway. The resulting changes in intracellularly available Ca^2+^ could contribute to the effects on vascular resistance.

Another non-renal parameter that could account for the MAP lowering action is a change in cardiac function, namely a decrease of the heart rate or of the stroke volume. No effect on heart rate was obtained with the drugs in the present study, excluding a mechanism involving a chronotropic action. Somova also failed to obtain a decrease of heart rate following 6-week intraperitoneal administration of OA in DSS rats [19]. In another study the same authors reported a 6% decrease of heart rate [33]. This bradycardic effect was however too modest to account for the marked (20–35%) decrease in MAP concurrently obtained in the same animals. In the absence of a change in heart rate, a decrease of cardiac output and of MAP can be brought about by a change in stroke volume. We were not able to measure stroke volume in the present setting. However, our preliminary experiments using isolated myocytes show that OA and derivatives do not decrease but rather tend to increase rat ventricular myocyte shortening. This suggests that a negative inotropic action, resulting in a decrease of stroke volume, is also excluded as mechanism for the hypotensive action of OA and its derivatives.

Hypertension is associated with oxidative stress which causes cell dysfunction because of increased production of reactive oxygen species (ROS). Among affected functions are those involving Na^+^ reabsorption by the kidney and its regulation by the renin-angiotensin-aldosterone [34]. Increased production of ROS such as superoxide anion due to decreased antioxidant defense systems causes rapid inactivation of nitric oxide (NO) signaling and bioavailability [35,36] with potential consequences on kidney function [37]. OA has been shown to cause release of NO via a Ca^2+^-independent increase in phosphorylation of NO synthase [29,31,32]. Since NO is known to relax vascular smooth muscle and to exert inhibitory effects on tubular Na^+^ reabsorption, the increased bioavailability of NO may play a role both for the blood pressure reduction and increased renal sodium excretion.

Our study investigated the oxidative status of kidney, liver and hearts harvested from untreated and OA-treated SHR and DSS animals at the end of the 9-week study period. Although the most direct approach for the assessment of lipid peroxidation is the quantification of the primary (hydroperoxides) products, these peroxides are labile and short-lived. Hence, detection of lipid peroxidation has relied largely on indirect methods, that is, analyses of secondary or end products derived from hydroperoxides such as malonyldialdehyde (MDA) [26]. We therefore determined the levels of lipid peroxidation expressed as thiobarbituric acid reactive substances (TBARS; MDA). We also determined oxidative protein damage as indicated by activities of antioxidant enzymes including superoxide dismutase (SOD) and glutathione peroxidase (GPx). The results show that OA-treated SHR and DSS animals have less abnormal to normal levels of MDA, SOD and GPx in comparison to untreated hypertensive rats. We speculate that antioxidant properties of OA could play a role in hypotensive mechanisms of this triterpene. How this can be related to nitric oxide (NO) [38,39] remains to be tested.

In conclusion, we provide the first report about hypotensive and natriuretic effects of novel OA derivatives, Me-OA and Br-OA. These derivatives possess more potent natriuretic effects in comparison to parent OA, mediated in part via the inhibition of proximal tubular reabsorption. This study also provides the first *in vivo* evidence that OA-mediated hypotensive effects are more marked, are correlated with more pronounced natriuretic effects and are associated with a reduction of high aldosterone levels in hypertensive animals. We also show that the antihypertensive action of OA are associated with a modulation of oxidative status in cardiac, renal and hepatic tissues in hypertensive animals.

### Limitations of the study

The observed correlation between changes in blood pressure and Na^+^ excretion does not prove a causal relation between the two, especially since we did not find any change in fluid elimination. Additional work is needed to further explore the mechanisms of action of OA and derivatives. The role of NO, a key regulator of vascular and renal functions, in mediating OA effects was not investigated in the present study. Additional studies on endocrine function may also be needed. The present results, by showing that OA decreased plasma aldosterone level, suggest that alterations in the renin-angiotensin-aldosterone system (RAAS) may be involved in the effect of the triterpene. Beside the aldosterone level, other components of the general and renal RAAS (such as expression of renin, angiotensin converting enzyme or angiotensinogen genes; levels of renin and angiotensin II and expression of their receptors in kidney; urinary aldosterone or angiotensinogen level; etc.) need to be evaluated. In addition the mechanisms by which OA modulates oxidative stress remain undefined. Recent studies have demonstrated that the activation of nuclear factor erythroid 2-related factor 2 (Nrf2) accounts for effects of OA on oxidative stress (Liu et al. Oleanolic acid co-administration alleviates ethanol-induced hepatic injury via Nrf-2 and ethanol-metabolizing modulating in rats [40]. Analysis of the Nrf2 level and of its target antioxidants was not carried out in the present study but would be useful to envisage.

## Supporting Information

S1 Fig *Syzygium aromaticum*-derived OA ^13^C- NMR spectra (A), ^1^H-NMR of Me-OA (B) and ^13^C- NMR spectrum of Br-OA (C). Structures were elucidated using NMR spectra recorded on a Bruker DRX-400 spectrometer.
(DOCX)

S2 Fig Data of GFR (A) and MAP (B) of control (untreated) rats and animals administered OA, Me-OA and Br-OA (90 μg h^-1^) during the 4 h experimental period. All drugs were administered for 1.5 h during the treatment period. Values are presented as means ± S.E.M. for each 30 min collection (n = 6 in each group).
(XLSX)

S3 Fig Data of urine flow (A) urinary Na^+^ excretion (B), FE_Na proximal_ tubule (C) FE_Li proximal_, and FE_Li distal_ (D) of control rats and animals infused OA, Me-OA and Br-OA. Drugs were administered for 1.5 h during the treatment period. Values are presented as means ± S.E.M. for each 30 min collection (n = 6 in each group).
(XLSX)

S4 Fig Data of the changes in MAP (A), urinary Na^+^ excretion (B); correlation between MAP and Na^+^ changes (C) in OA or derivative-administered animals obtained during the 1.5 h treatment period. Values are presented as means ± S.E.M. (n = 6 in each group).
(XLSX)

S5 Fig Data obtained following the administration of various doses of OA (30, 60, 120 mg kg^-1^) on MAP Wistar (A), SHR (B) and DSS (C) rats over a 9-week experimental period. Values are presented as weekly means ± S.E.M. (n = 6 in each group).
(XLSX)

S6 Fig Data obtained following the administration of various doses of OA (30, 60, 120 mg kg^-1^) on 24 h urine flow (A-C) and Na^+^ excretion (D-F) rates with control Wistar, SHR and DSS animals over a 9-week experimental period. Values are presented as weekly means ± S.E.M. (n = 6 in each group).
(XLSX)

S7 Fig Data obtained following the administration of various doses of OA (30, 60, 120 mg kg^-1^) of changes in MAP (A) and urinary Na^+^ excretion rate (B) on the 9^th^ week of the study. Correlation of MAP and Na^+^ (C) changes in conscious Wistar, SHR and DSS rats. Values are presented as weekly means ± S.E.M. (n = 6 in each group).
(XLSX)
