# Supplementary figures and images for: Changes in Renal Function and Oxidative Status Associated with the Hypotensive Effects of Oleanolic Acid and Related Synthetic Derivatives in Experimental Animals
Source: PLoS One. 2015 Jun 5;10(6):e0128192. doi: 10.1371/journal.pone.0128192 (PMC4457832; doi:10.1371/journal.pone.0128192)

**A - OLEANOLIC ACID**

**B- METHYL ESTER OF OA (ME-OA)**


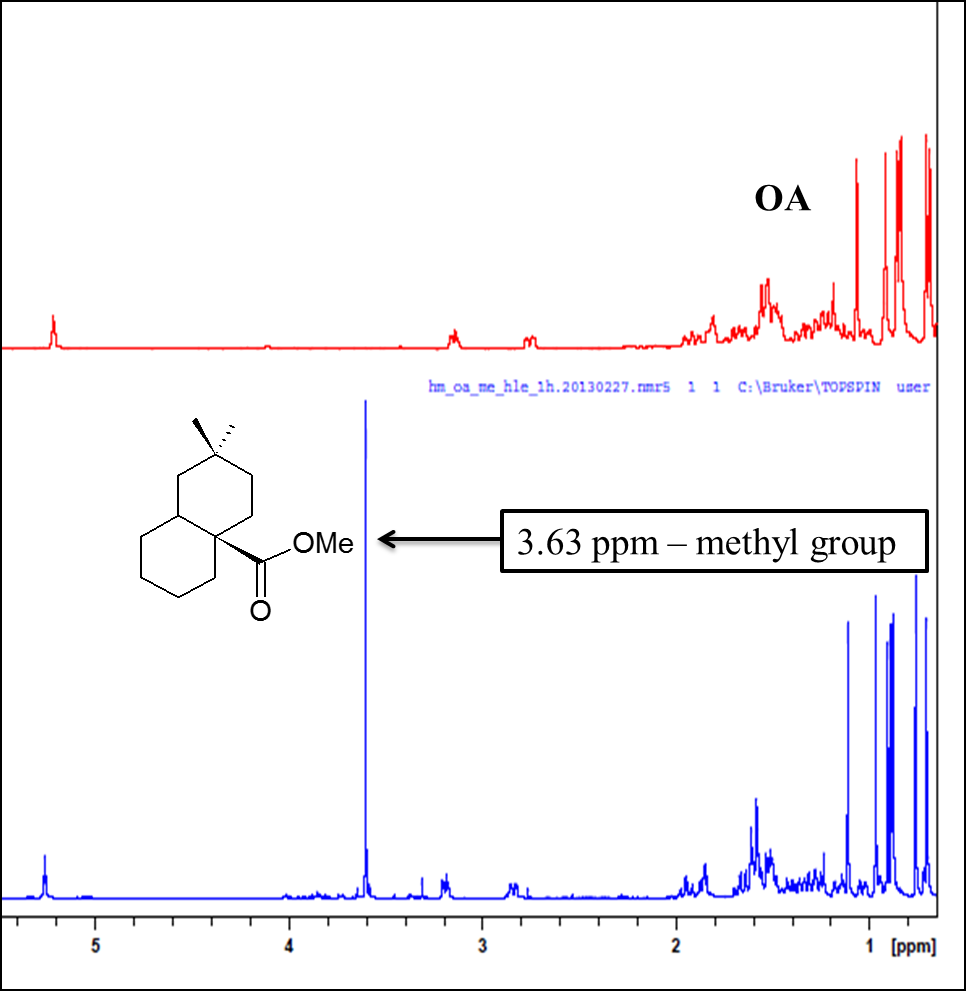


**C- BROMINATED DERIVATIVE OF OA (BR-OA)**

Supplement: S1 Fig — Structures were elucidated using NMR spectra recorded on a Bruker DRX-400 spectrometer. (DOCX) [file pone.0128192.s001.docx]
